# Robotic-assisted bronchoscopy combined with digital tomosynthesis for pulmonary nodules characterization: a single center experience

**DOI:** 10.3389/fmed.2026.1864679

**Published:** 2026-07-21

**Authors:** Flavio Marco Mirabelli, Emma Repaci, Gian Piero Bandelli, Thomas Galasso, Martina Ferioli, Marco Ferrari, Filippo Natali, Tommaso Abbate, Teresa Calari, Stefania Damiani, Francesca Giunchi, Mattia Riefolo, Lorenzo Scrofani, Annalisa Altimari, Elisa Gruppioni, Daniela Di Luca, Federica Pasquali, Piero Candoli

**Affiliations:** 1 Respiratory Medicine and Thoracic Endoscopy Unit, Ospedale Regina Apostolorum, Albano Laziale, Rome, Italy; 2 Department of Internal Medicine, Ospedale Policlinico Casilino, Rome, Italy; 3 Interventional Pulmonology Unit, IRCCS Azienda Ospedaliero-Universitaria di Bologna, Bologna, Italy; 4 Pathological Anatomy and Histology Unit, IRCCS Azienda Ospedaliero-Universitaria di Bologna, Bologna, Italy; 5 Solid Tumor Molecular Pathology Laboratory, IRCCS Azienda Ospedaliero-Universitaria di Bologna, Bologna, Italy; 6 Cardio-thoracic and Vascular Anesthesia and Intensive Care Unit, IRCCS Azienda Ospedaliero-Universitaria di Bologna, Bologna, Italy; 7 Clinical Engineering Division, IRCCS Azienda Ospedaliero-Universitaria di Bologna, Bologna, Italy

**Keywords:** AI–tomography, artificial intelligence, augmented fluoroscopy, diagnostic yield, interventional pulmonology, LungVision, peripheral pulmonary lesions, robotic-assisted bronchoscopy

## Abstract

**Introduction:**

Diagnostic work-up of peripheral pulmonary lesions (PPLs) remains a challenge in interventional pulmonology. Conventional bronchoscopy and trans-thoracic needle aspiration (TTNA) often entail limitations in accuracy or safety. Robotic-assisted bronchoscopy with shape-sensing technology (ssRAB) combined with artificial intelligence (AI)—aided augmented fluoroscopy (LungVision system) represents an innovative approach to enhance lesion localization and diagnostic yield for small or otherwise hard-to-reach lesions while maintaining the safety profile of the procedure.

**Materials and methods:**

This retrospective observational study included all procedures performed under general anesthesia using the ssRAB (Ion™ robotic platform) in combination with C-arm based computed tomography (LungVision System) at the Interventional Pulmonology Unit of IRCCS Azienda Ospedaliero-Universitaria, Policlinico Sant’Orsola, Bologna (Italy), between December 2024 and September 2025. Overall, 82 patients with 94 sampled pulmonary lesions were included; in 12 patients, two distinct nodules were sampled during the same bronchoscopic procedure. Lesions features, procedural characteristics, diagnostic yield and complications were collected and statistically analyzed. Diagnostic yield was defined according to the 2024 Delphi consensus and STARD 2015 guidelines.

**Results:**

This study included 82 patients and 94 sampled pulmonary nodules. The median size of the lesion was 14 mm (IQR 11–18 mm). Target lesions were identified by radial endobronchial ultrasound (r-EBUS) in 80% of cases. A definitive cyto-histologic diagnosis was achieved in 74.5% of cases. Lesions ≥10 mm yielded a 79.2% diagnostic rate, versus 59.1% for smaller nodules. Diagnostic success was independently predicted by upper lobe location (OR 3.28; *p* = 0.031) and lesion visibility on CABT (OR 3.22; *p* = 0.043). The overall complication rate was 3.3% (three pneumothorax cases; no major bleeding). The median procedure time was 34.5 min, and most procedures were performed in a day-hospital setting.

**Discussion and conclusions:**

The integration of robotic-assisted bronchoscopy with the LungVision system exhibited high diagnostic efficacy and a strong safety profile when sampling peripheral lung lesions. The synergy of real-time AI aided augmented fluoroscopy and robotic technologies facilitates precise lesion targeting, even within anatomically complex areas. However, follow-up data were not available for non-diagnostic cases; therefore, false-negative rates, sensitivity, and diagnostic accuracy could not be assessed. Given the significant cost associated with this procedure, future prospective studies are warranted to further validate these results and refine patient selection criteria to optimize its clinical application.

## 1 Introduction

With the widespread implementation of low-dose computed tomography (LDCT) screening programs, the detection of peripheral pulmonary lesions (PPLs) has increased markedly, with many lesions identified in asymptomatic individuals (1, 2). While earlier diagnosis has substantially improved lung cancer outcomes, it has also heightened the demand for minimally invasive diagnostic techniques capable of providing accurate histological and molecular characterization (3, 4).

Conventional diagnostic approaches—such as standard bronchoscopy and transthoracic needle aspiration (TTNA)—have important limitations. Bronchoscopy is recognized for its favorable safety profile (5); however, its diagnostic yield is often suboptimal, particularly for small and/or peripherally located lesions (6). Conversely, TTNA offers high diagnostic accuracy but is associated with a higher complication rate, most notably pneumothorax, which may necessitate hospital admission (7, 8).

Recent technological advances have reshaped bronchoscopic diagnostics. Adjuncts including virtual bronchoscopy, electromagnetic navigation, radial endobronchial ultrasound (r-EBUS), and cone-beam CT have progressively improved access to peripheral airways. Among these innovations, robotic-assisted bronchoscopy (RAB) represents a major development, offering enhanced stability, more precise navigation, and more consistent performance, even in anatomically complex regions.

The Ion™ robotic platform employs shape-sensing technology to provide real-time feedback on catheter position, enabling accurate navigation without reliance on electromagnetic fields. When combined with digital tomosynthesis (DT) systems such as LungVision™, this approach allows real-time visualization of target lesions, thereby reducing CT-to-body divergence and improving targeting accuracy. Notably, LungVision provides real-time three-dimensional reconstruction of pulmonary anatomy through integrated artificial intelligence, generating tomographic images from standard multiplanar X-ray acquisitions.

Despite increasing adoption, real-world evidence on the combined use of RAB and DT remains limited. This study aims to assess the diagnostic performance, safety, and clinical feasibility of this integrated approach in a real-world cohort of patients undergoing evaluation for peripheral pulmonary lesions.

## 2 Materials and methods

### 2.1 Study design and population

This retrospective observational study was conducted at the Interventional Pulmonology Unit of a tertiary referral center between December 2024 and September 2025. The cohort comprised adult patients who underwent robotic-assisted bronchoscopy (RAB) for the diagnostic assessment of peripheral pulmonary lesions (PPLs) and pulmonary nodules.

Eligible patients were included on the basis of clinical and radiological evaluation if they had one or more peripheral pulmonary lesions identified on chest computed tomography (CT) and met criteria for bronchoscopic sampling as determined by multidisciplinary review. The study population comprised all procedures performed using the Ion™ robotic-assisted bronchoscopy system (Intuitive Surgical, Sunnyvale, CA, USA) in conjunction with augmented fluoroscopy with C-arm based computed tomography provided by the LungVision™ system (Body Vision Medical, Israel).

The study was conducted in accordance with the Declaration of Helsinki and was approved by the local institutional review board. Due to the retrospective observational design, the requirement for written informed consent was waived according to institutional policies.

### 2.2 Pre-procedural assessment

All patients underwent comprehensive pre-procedural assessments, which included collection of clinical history, physical examination, pulmonary function testing when appropriate and review of high-resolution chest CT scans. Thin-slice CT imaging (≤1.5 mm thickness) was required for procedural planning and navigation.

Lesions were evaluated based on maximal axial diameter, lobar and segmental location, presence of a CT bronchus sign and CT density (solid or subsolid appearance).

Pre-procedural anesthesiologic evaluation was performed in all patients and anesthetic risk was stratified using the American Society of Anesthesiologists (ASA) physical status classification. Anticoagulant and antiplatelet therapies were managed according to international guidelines and protocols (9).

### 2.3 Procedural setting and anesthesia

All procedures were performed in a dedicated interventional pulmonology suite equipped with fluoroscopy and advanced imaging systems. Patients underwent general anesthesia with orotracheal intubation to ensure optimal airway control, minimize patient movement, and facilitate prolonged and precise navigation.

Mechanical ventilation was provided using volume-controlled ventilation with lung-protective settings. Attention was paid to minimizing atelectasis, including careful preoxygenation strategies with avoidance of excessive fraction of inspired oxygen (FiO₂) and the use of high PEEP (8–12 cmH_2_O) whenever feasible.

Continuous monitoring involved electrocardiography, non-invasive blood pressure measurement, pulse oximetry, assessment of hypnotic depth through bispectral index (BIS) and evaluation of neuromuscular blockade and recovery using train-of-four (TOF). A dedicated anesthesiology team remained present throughout the procedure.

### 2.4 Robotic-assisted bronchoscopy system

All procedures were performed using the Ion™ Endoluminal System (Intuitive Surgical), a robotic-assisted bronchoscopy platform based on shape-sensing technology (ss-AB). The system consists of a flexible robotic catheter with a 3.5 mm outer diameter and a 2.0 mm working channel with an integrated vision probe with a 1.7 mm diameter, a robotic drive unit with a control interface enabling precise advancement and articulation, a planning workstation (PlanPoint™) used for pre-procedural route planning. The shape-sensing technology of the catheter enables real-time tracking of its position and orientation allowing accurate navigation even in anatomically complex regions.

**Figure 1:**
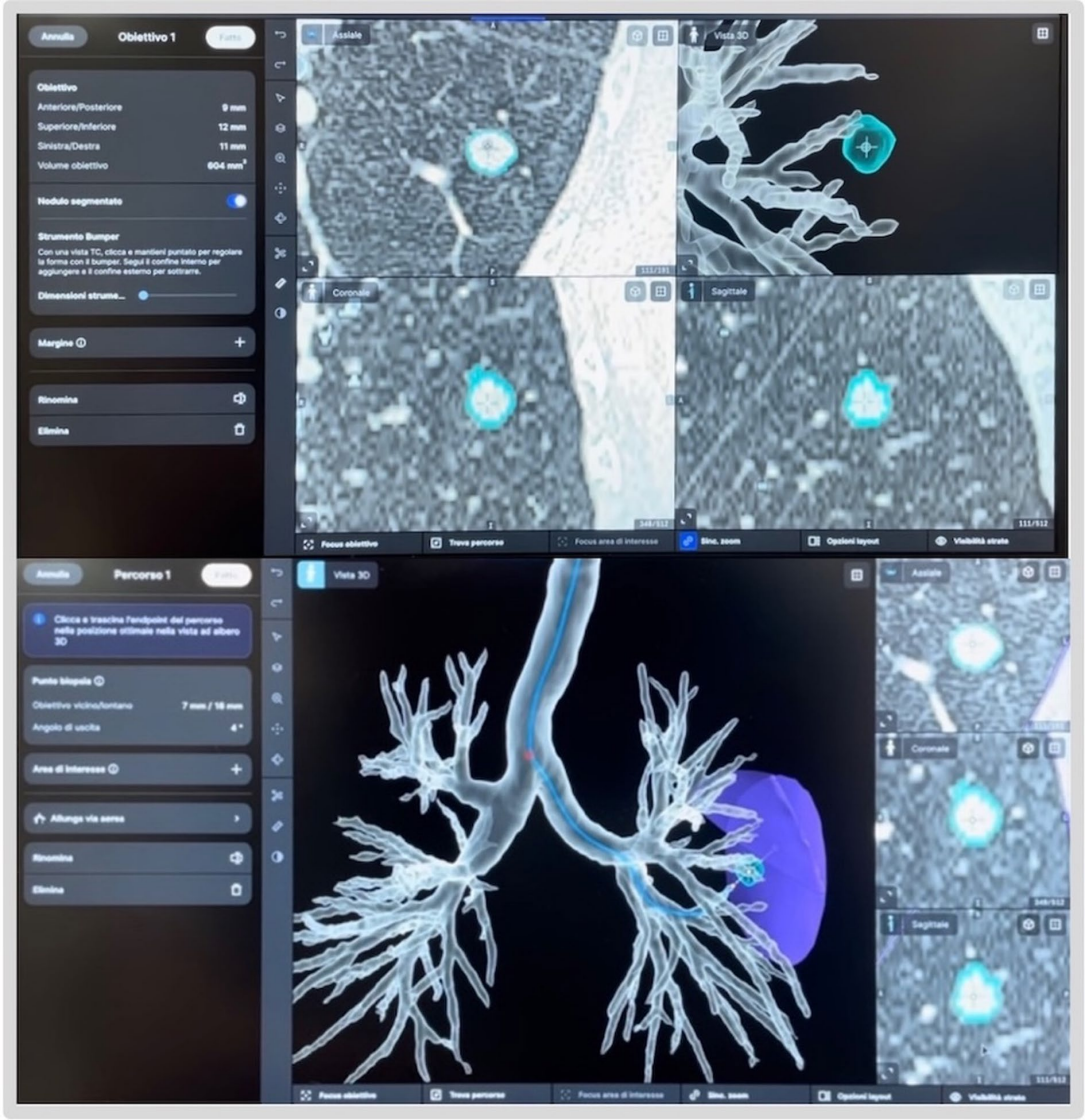
Pre-procedural planning on the PlanPoint™ System. Three-dimensional reconstruction of the bronchial tree and pre-procedural planning. The target lesion and bronchial pathway are marked in blue and the anatomical limits (pleura) are shown in purple.

High-resolution CT scans were used for three-dimensional reconstruction of the bronchial tree and pre-procedural planning. Target lesion was identified manually and optimal bronchial pathway chosen by the operator based on airway accessibility, lesion characteristics and proximity to vascular structures and pleura (Figure 1). Following induction of anesthesia and initial airway inspection, the robotic bronchoscope was introduced through the endotracheal tube. Registration between the virtual airway model and the patient’s anatomy was achieved through sequential alignment of anatomical landmarks, such as trachea and main bronchi, ensuring spatial accuracy and minimizing CT-to-body divergence. Robotic navigation toward the target lesion was then performed using the control interface, with continuous correlation between endoscopic visual feedback and the virtual navigation map.

### 2.5 Integration of digital Tomosynthesis (LungVision™ system)

DT utilizing the LungVision™ system was conducted to improve real-time lesion localization and address CT-to-body divergence.

The system integrates pre-procedural CT data with live fluoroscopic images through artificial intelligence–based algorithms. By generating a three-dimensional reconstruction (C-arm–based tomography) from fluoroscopic images acquired via a C-arm, it allows continuous visualization of the lesion and its spatial relationship to biopsy tools. This facilitates accurate targeting, particularly when dealing with small, subsolid or poorly visible nodules.

**Figure 2:**
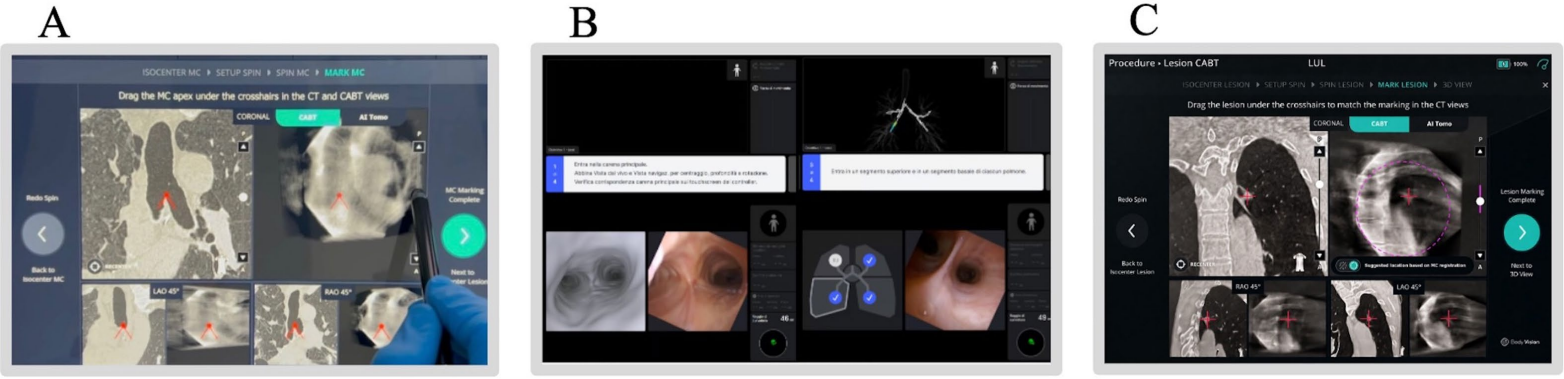
Workflow of integration between ssRAB and Digital Tomosynthesis (LungVision™ System): **(A)** MC centering, **(B)** Registration, **(C)** Lesion target centering. **(A)** MC centering: the LungVision™ cursor is centered on the carina. **(B)** Registration: alignment of the patient anatomy with the virtual lung map. **(C)** Lesion target centering: the target lesion is centered in the system.

During the procedural phase, the C-arm is positioned at the level of the carina and rotated from +30° to −30° (first spin) to obtain a three-dimensional reconstruction of the carina. Its position must be matched on both CT and CABT images, performing its “centering” (10). This step occurs before registration with the robotic system (ssRAB). Subsequently, the software indicates how the C-arm position should be adjusted along the three axes to place the target lesion at the center of the fluoroscopic beam. After ssRAB registration has been completed, lesion centering is achieved through an additional 60° rotation of the C-arm around the target lesion (second spin), with final confirmation on CABT images (10) (Figure 2).

### 2.6 Lesion localization and sampling strategy

**Figure 3:**
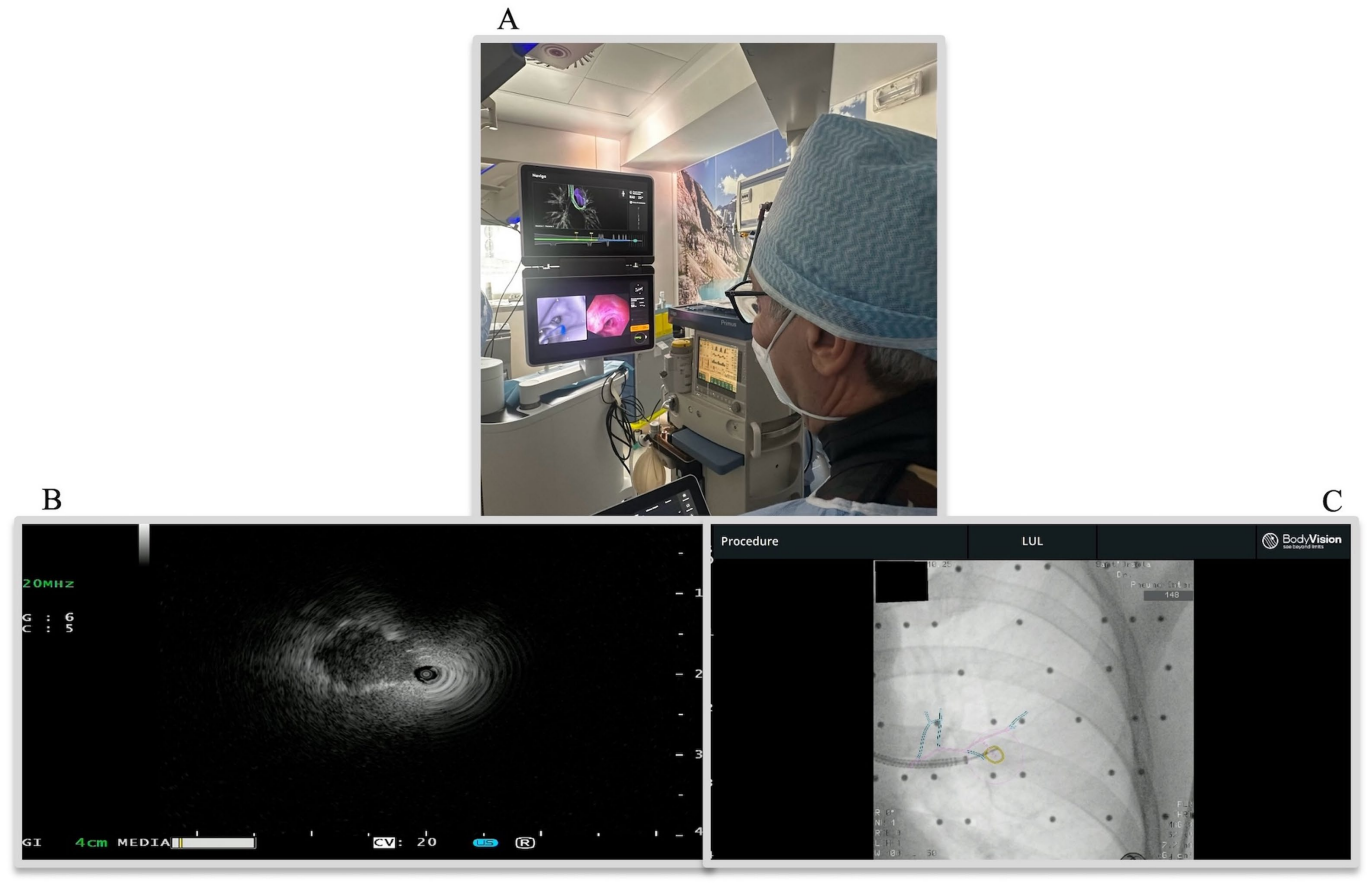
Lesion localization during the procedure with RAB and LungVision™ System: **(A)** Robotic navigation, **(B)** rEBUS, **(C)** Augmented fluoroscopy (LungVision™ System). **(A)** Robotic navigation: the robotic bronchoscope follows the planned path to the target lesion. **(B)** rEBUS: endobronchial ultrasound imaging confirms the location and characteristics of the lesion. **(C)** Augmented fluoroscopy (LungVision™ System): real-time augmented fluoroscopy visualizes the lesion during navigation.

Lesion localization was confirmed using a multimodal approach combining robotic navigation, augmented fluoroscopy and radial endobronchial ultrasound (r-EBUS; Figure 3). Radial EBUS findings were classified according to established patterns (concentric, eccentric, adjacent or absent signal) (11, 12). Sampling was initiated once lesion localization was deemed satisfactory by at least one imaging modality.

Sampling techniques included: transbronchial biopsy forceps (PENTAX), transbronchial needle aspiration (TBNA/FNA) with the 21G ION needle, transbronchial lung cryobiopsy (TBLC) with 1.1 mm cryoprobe (ERBE) or a combination of multiple biopsy strategies. Final tool selection remained at the operator’s discretion but followed institutional practice based on lesion size, anatomical location, radiological characteristics, presence of a CT bronchus sign, r-EBUS findings, and intra-procedural assessment of lesion accessibility and tool positioning. Multiple samples were obtained from each lesion whenever feasible to maximize diagnostic yield.

### 2.7 Rapid on-site evaluation

**Figure 4:**
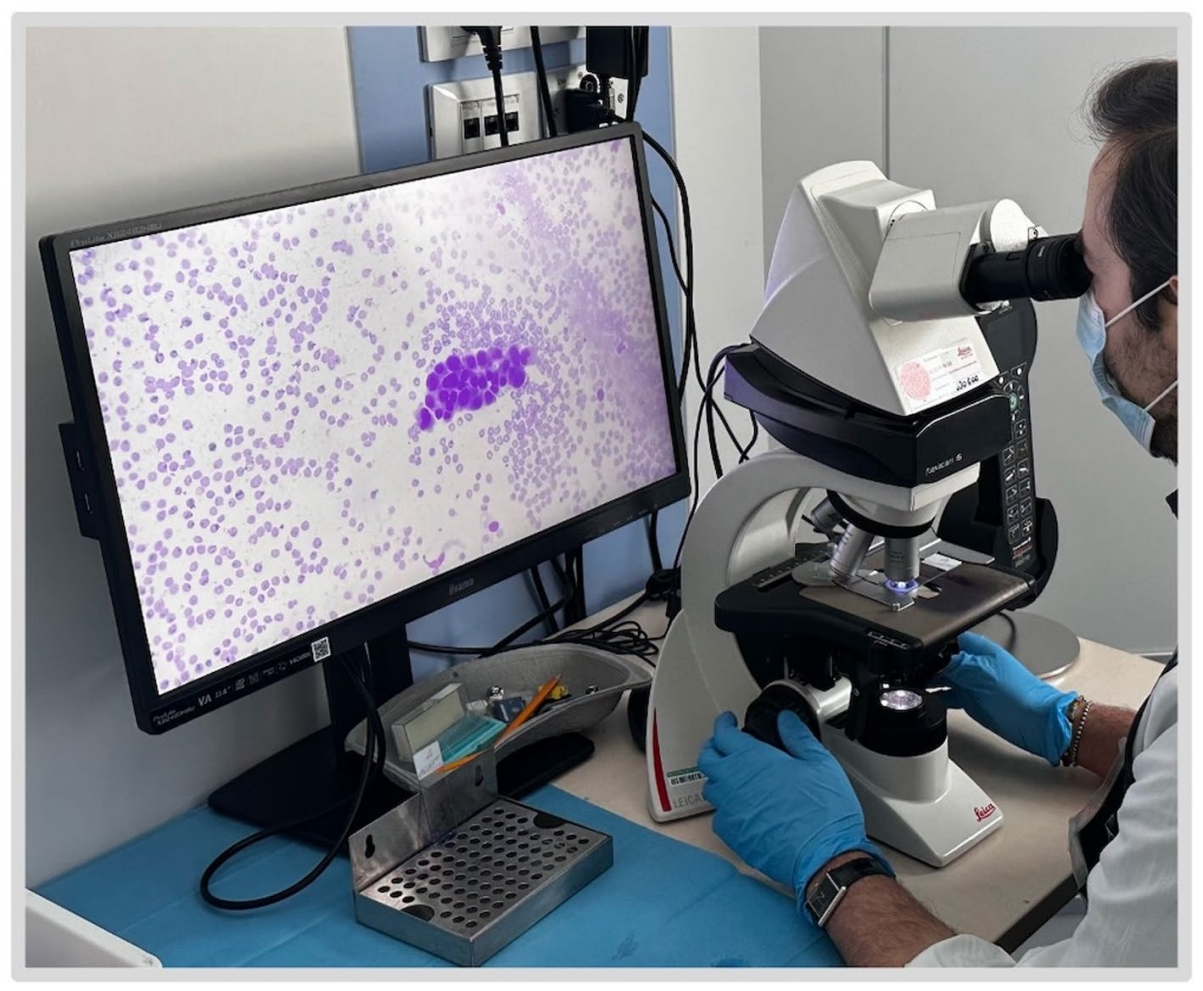
ROSE performed by the cytopathologist during the procedure. Rapid on-site evaluation (ROSE) allows real-time cytological assessment of sample adequacy.

Rapid on-site evaluation (ROSE) was performed by an experienced cytopathologist to assess specimen adequacy in real time (Figure 4). ROSE findings guided the number of sampling passes and informed decisions regarding the need for additional biopsies or alternative sampling techniques.

### 2.8 Post-procedural management and safety assessment

Following the procedure, patients were monitored in a recovery area until full anesthetic recovery. Adverse events were recorded and classified as minor or major. Major complications included pneumothorax requiring chest tube placement, significant bleeding (Nashville scale > 2), respiratory failure or hemodynamic instability. Minor complications included self-limiting bleeding or transient hypoxemia.

### 2.9 Outcome measures

The primary outcome was strict diagnostic yield, defined as the proportion of procedures resulting in a definitive pathological diagnosis, according to the 2024 Delphi consensus (13) and STARD 2015 (14) criteria. Cases showing atypia, non-specific acute or chronic inflammation, normal lung parenchyma, or insufficient material were classified as non-diagnostic according to the 2024 Delphi consensus definition.

Secondary outcomes included: diagnostic yield stratified by lesion size and location, total procedural time (measured from bronchoscope insertion to completion of sampling), registration and navigation time, sampling time and complication rate.

### 2.10 Statistical analysis

Continuous variables were reported as median and interquartile range (IQR), while categorical variables were expressed as absolute numbers and percentages. Comparisons between groups were performed using the Mann–Whitney U test for continuous variables and the χ^2^ or Fisher’s exact test for categorical variables, as appropriate.

Univariate and multivariate logistic regression analyses were conducted to identify predictors of diagnostic success. Univariate analyses were performed using χ^2^ tests or simple logistic regression to assess the association between variables of interest and diagnostic yield. Variables showing statistical significance or a positive trend (*p* < 0.10) in univariate analysis were selected for inclusion in the multivariate model. Multivariate analysis was conducted using binary logistic regression, including a maximum of six independent variables in accordance with the rule of 10 events per variable (15), and model performance was assessed using McFadden’s R^2^ (R^2^McF).

Results were expressed as odds ratios (ORs) with 95% confidence intervals (CIs). A *p*-value <0.05 was considered statistically significant.

As a sensitivity analysis, a mixed-effects logistic regression model including patient ID as a random intercept was performed to account for potential clustering of lesions within the same patient.

Statistical analyses were performed using Jamovi (version 2.7.6.0)

## 3 Results

### 3.1 Study population

The study was conducted over a 10-month period and included 94 pulmonary lesions sampled in 82 patients (Table 1). Twelve patients underwent sampling of two distinct pulmonary lesions during the same procedural session. The study population was evenly distributed by sex (50% male and 50% female), with a median age of 71 years. Most patients were normal weight, with a median BMI of 25 kg/m^2^, and obesity was observed in a minority of cases (14.6%). More than half of the patients had a history of smoking and approximately one quarter were active smokers at the time of the study. A substantial proportion of patients (39%) had a prior history of malignancy, including both pulmonary and extrapulmonary cancers. From an anesthesiologic standpoint, the majority of patients were classified as intermediate risk, with most falling into ASA classes II and III, while a smaller proportion were classified as high anesthetic risk (ASA IV).

**Table 1:** Population demographic and clinical characteristics.

Population characteristics (82 patients)			
Variable	Category/Statistic	Value/Number	Percentage/IQR
Gender	Male	41	50.0%
	Female	41	50.0%
Age in years	Median, IQR	71	63–76
BMI (kg/m^2^)	Median, IQR	25	23–29
Smoking history	Current smoker	19	23.2%
	Former smoker	46	56.1%
	Never smoker	17	20.7%
Pack/year	Median, IQR	36	16–50
Cancer history		32	39%
Lung cancer	Adenocarcinoma	4	12.5%
	Squamous cell carcinoma	1	3.1%
	Other	0	0.0%
Other cancer	Gastrointestinal	6	18.8%
	Breast	5	15.6%
	Kidney	2	6.3%
	Skin	3	9.4%
	Other	14	43.8%
ASA score	1	1	1.2%
	2	27	31.8%
	3	45	52.9%
	4	9	10.6%

IQR, interquartile range; DS, standard deviation; BMI, Body Mass Index; ASA score, American Society of Anesthesiologists score.

### 3.2 Lesion characteristics

A total of 94 pulmonary lesions were evaluated. Nodule characteristics are summarized in Table 2.

**Table 2:** Nodule characteristics.

Nodule characteristics — total nodules (*n* = 94)			
Variable	Category/Statistic	Value/Number	Percentage/IQR
Dimension	Mean diameter, mm — median, IQR	14	11–18
	Volume (mm^3^) — median, IQR	1148	571–2,527
Type	Solid	57	60.6%
	Part-solid	21	22.3%
	Pure ground glass	12	12.8%
	Cavitary	4	4.3%
Location, regional	Central	8	8.5%
	Middle	23	24.5%
	Peripheral	63	67.0%
Location, anatomical	RUL	33	35.1%
	RML	5	5.3%
	RLL	17	18.1%
	LUL	21	22.3%
	LLL	18	19.1%
Distance to pleura, mm	Median, IQR	19	14–29
CT bronchus-sign	Present	72	76.6%
	Absent	22	23.4%
Malignancy probability, Brock Model	Median, IQR	35%	18–52%

IQR, interquartile range; RUL, Right upper lobe; RML, Right middle lobe; RLL, Right lower lobe; LUL, Left upper lobe; LLL, Left lower lobe.

The median diameter of the lesions was 14 mm (interquartile range [IQR]: 11–18 mm). The majority of lesions were situated in the upper lobes (63%), with a modest predominance in the right lung (58.5%). A substantial proportion of lesions were sub centimetric or close to the 10 mm threshold. Most cases involved solid nodules (60.6%), whereas subsolid lesions were part-solid (22.3%) and ground-glass opacities (12.8%).

A bronchus sign on pre-procedural CT imaging was identified in a significant proportion of cases (76.6%), although its presence varied depending on lesion size and anatomical location. Most lesions were peripherally located (67%), while a smaller proportion were situated in central and intermediate regions of the lung (8.5 and 24.5%, respectively).

### 3.3 Procedural characteristics and technical performance

The total median procedural time was 34.5 min (IQR: 26–48 min; Table 3).

**Table 3:** Diagnostic tools, lesion-level sampling characteristics, and session-level procedural variables for sampled lesions.

Diagnostic tools, sampling characteristics and procedural variables — ssRAB sampled nodules (*n* = 94)			
Variable	Category/Statistic	Value/Number	Percentage/IQR
Timing, min	Total procedure time — median, IQR	34.5	26–48
	Registration and navigation — median, IQR	12	8–20
	Sampling — median, IQR	14	8–20
X-ray exposure, sec	Median, IQR	344	231–479
X-ray exposure, dose, cGycm^2^	Median, IQR	294	192–430
CABT visibility	Present	70	74.5%
r-EBUS	Present	76	80.9%
	Concentric	11	14.5%
	Eccentric	46	60.5%
	Adjacent	19	25.0%
Sampling tool	Forceps	82	87.2%
	FNA/TBNA	24	25.5%
	Cryoprobe	28	29.8%
EBUS-TBNA	Total	19	23.2%
PEEP, cmH_2_O	12	21	22.0%
	10	54	57.0%
	8	19	20.0%
Complications	Total	3	3.3%

IQR, interquartile range; ssRAB, shape sensing robotic assisted bronchoscopy; LV, LungVision; CABT, C-arm based tomography; r-EBUS, radial endobronchial ultrasound; EBUS-TBNA, conventional linear endobronchial ultrasound-guided transbronchial needle aspiration (for mediastinal staging); PEEP, positive end-expiratory pressure; FNA/TBNA, fine needle aspiration/ transbronchial needle aspiration performed with the ION 21G needle. Targeting, imaging, and sampling variables are reported at the sampled-lesion level; total procedure time, X-ray exposure, and complications refer to the bronchoscopic session in which the lesion was sampled.

Radial endobronchial ultrasound (r-EBUS) was used to confirm lesion localization. A concentric or eccentric ultrasound signal was obtained in approximately 80% of procedures, allowing confident confirmation of lesion proximity prior to sampling.

Tissue sampling was performed using different tools, most frequently forceps, followed by cryoprobe and TBNA needle, alone or in combination. The overall complication rate was 3.3%, with three post-procedural pneumothoraces and no significant bleeding events; two pneumothoraces required chest tube placement and monitored hospitalization.

No procedures were discontinued due to anesthetic or technical complications.

### 3.4 Diagnostic yield

A definitive cyto-histological diagnosis was achieved in 70 of 94 sampled lesions, corresponding to an overall diagnostic yield of 74.5% (strict diagnostic definition). The remaining 24 samples were classified as non-diagnostic according to the 2024 Delphi consensus definition, including cases with atypia, non-specific acute or chronic inflammation, normal lung parenchyma, or insufficient material.

**Figure 5:**
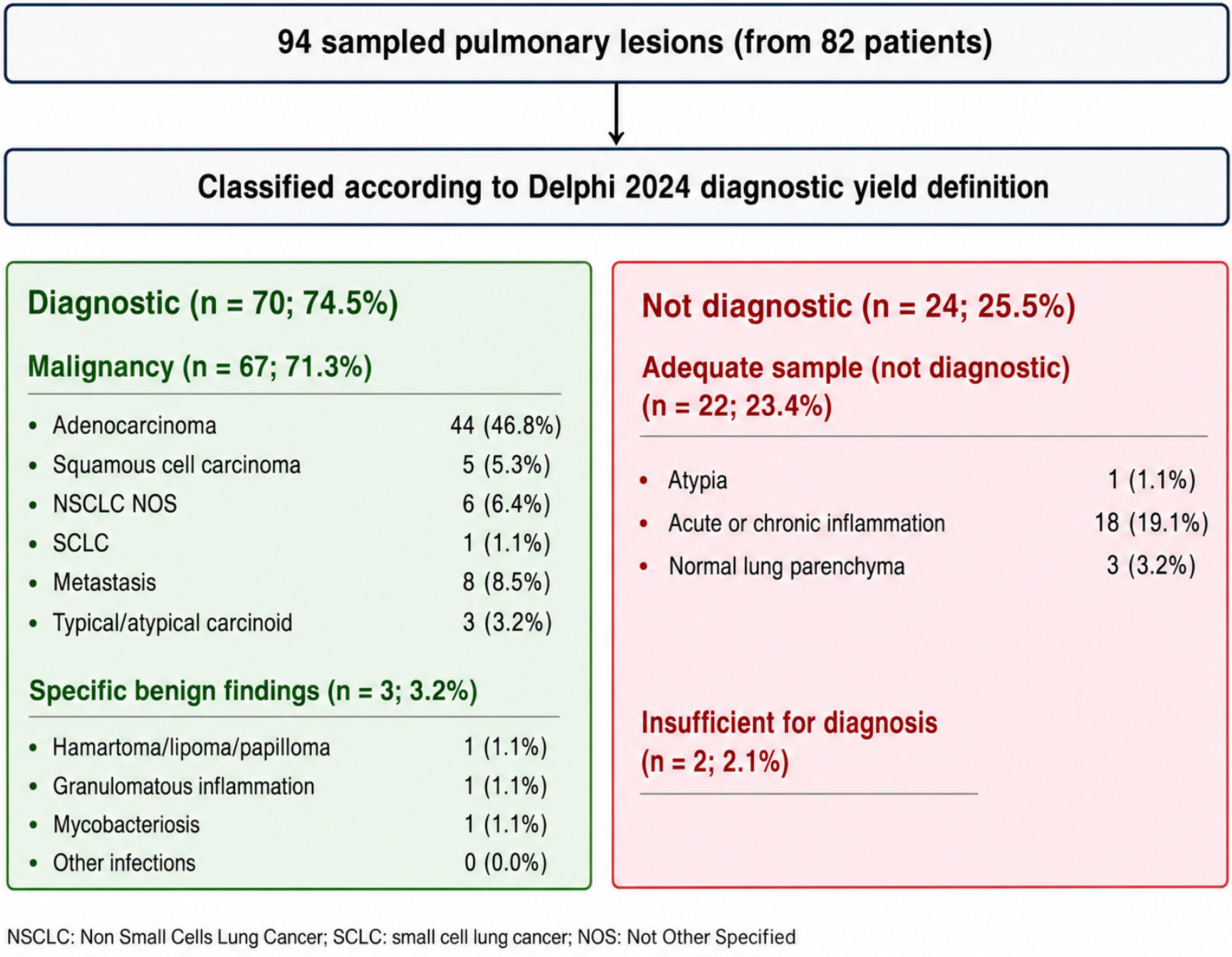
Classification of sampled lesions according to the 2024 Delphi consensus definition of diagnostic yield. Among 94 sampled pulmonary lesions from 82 patients, 70 were classified as diagnostic and 24 as non-diagnostic. Non-diagnostic cases included both adequate but non-diagnostic samples (atypia, non-specific acute or chronic inflammation, and normal lung parenchyma) and insufficient samples for diagnosis.

A flow diagram summarizing the classification of all 94 sampled lesions according to the Delphi criteria is provided in Figure 5.

When stratified by lesion size, diagnostic yield was significantly higher in lesions measuring ≥10 mm (79.2%) compared with lesions <10 mm (59.1%).

Among the 16 lesions measuring >20 mm in maximal diameter, a definitive diagnosis was achieved in 14 cases, corresponding to a diagnostic yield of 87.5%.

Lesions located in the upper lobes demonstrated a higher diagnostic yield compared with middle and lower lobe lesions (81.4% vs. 62.9%). Similarly, lesions with a visible bronchus sign on pre-procedural CT imaging were associated with improved diagnostic performance (78.2% vs. 56.2%).

As summarized in Table 4, histopathological diagnoses encompassed both malignant and specific benign conditions. Malignant diagnoses accounted for 67 of 94 lesions (71.3%), with non–small cell lung cancer being the most frequent category; adenocarcinoma was the most common histological subtype, representing 44 of 94 lesions (46.8%) and 65.7% of malignant diagnoses. Specific benign diagnoses accounted for 3 of 94 lesions (3.2%) and included benign tumors, infectious diseases, and granulomatous inflammation.

**Table 4:** ssRAB + LungVision diagnostic performance.

Pathology report — Total biopsies (*n* = 94)			
Diagnostic category	Pathological finding / Subcategory	Number	Percentage
Diagnostic	Total	70	74.5%
Malignancy	Total	67	71.3%
	NSCLC NOS	6	6.4%
	Adenocarcinoma	44	46.8%
	Squamous cell carcinoma	5	5.3%
	SCLC	1	1.1%
	Metastasis	8	8.5%
	Typical/atypical carcinoid	3	3.2%
Specific benign findings	Total	3	3.2%
	Hamartoma/lipoma/papilloma	1	1.1%
	Granulomatous inflammation	1	1.1%
	Mycobacteriosis	1	1.1%
	Other infections	0	0.0%
Not diagnostic	Total	24	25.5%
Adequate sample, not diagnostic	Atypia	1	1.1%
	Acute or chronic inflammation	18	19.1%
	Normal lung parenchyma	3	3.2%
Insufficient for diagnosis	Total	2	2.1%

NSCLC, Non Small Cells Lung Cancer; SCLC, small cell lung cancer; NOS, Not Other Specified. Diagnostic and non-diagnostic classifications were assigned according to the 2024 Delphi consensus definition of diagnostic yield.

Adequate tissue for molecular analysis by next-generation sequencing (NGS) was obtained in 9 of 14 cases in which molecular testing was requested, enabling comprehensive histological and molecular characterization for clinical decision-making. In the remaining 5 cases, NGS could not be successfully performed because of insufficient tumor cellularity and/or inadequate nucleic acid quality.

### 3.5 Predictors of diagnostic success

Univariate and multivariate logistic regression analyses were performed to identify factors associated with diagnostic success (Tables 5, 6).

**Table 5:** Univariate logistic regression for ssRAB and LungVision (DT).

Variable	Categories	% Diagnostic	Test	*p*-value
Diameter (mm)	-	-	-	0.072
Diameter ≥10 mm	≥10 vs <10	79.2% vs 59.1%	χ^2^ = 3.57	**0.059**
Volume (mm^3^)	-	-	-	0.172
Volume ≥ median value	≥1150 vs <1150 (mm^3^)	83.0% vs 66%	χ^2^ = 3.58	**0.058**
Nodule type	solid vs subsolid	73.7% vs 75.7%	χ^2^ = 0.05	0.829
CT bronchus-sign	absent/present	59.1% vs 79.2%	χ^2^ = 3.57	**0.059**
CABT visibility	not visible/visible	54.5% vs 80.6%	χ^2^ = 6.00	**0.014**
r-EBUS	identified/not identified	78.2% vs 56.2%	χ^2^ = 3.37	0.067
CT location	central/middle/peripheral	75% vs 74% vs 74.6%	χ^2^ = 0.005	0.997
Anatomical Location	Upper vs lower lobes	81.4% vs 62.9%	χ^2^ = 3.95	**0.047**
PEEP (cmH₂O)	8 / 10 / 12	73.7% vs 81.5% vs 57.2%	χ^2^ = 4.72	0.094
Cryobiopsy	no / yes	75.0% vs 73.3%	χ^2^ = 0.03	0.863

CT, Computerized Tomography; CABT, C-arm Based Tomography; r-EBUS, radial endobronchial ultrasound; PEEP, Positive end-Expiratory Pressure.

Bold values indicate statistically significant associations (*p* < 0.05).

**Table 6:** Multivariate analysis for ssRAB and LungVision (DT).

Predictors	SE	Odds Ratio (OR)	95% CI	*p*-value
Positive r-EBUS	0.662	2.804	0.77–10.27	0.119
CABT visibility	0.577	3.223	1.04–9.94	**0.043**
Upper Lobes Location	0.550	3.281	1.12–9.65	**0.031**
Bronchus- sign	0.586	1.863	0.59–5.87	0.288
Diameter ≥10 mm	0.671	2.023	0.54–7.53	0.294
Volume > median value	0.661	1.554	0.43–5.68	0.505

CABT, C-arm Based Tomography; r-EBUS, radial endobronchial ultrasound; OR, odds ratio; 95% CI, 95% confidence interval.

Bold values indicate statistically significant associations (*p* < 0.05).

In univariate analysis, nodule diameter and volume were not significantly associated with diagnostic yield when analyzed as continuous variables. When dichotomized according to median cutoffs, both larger diameter (≥10 mm) and higher volume lesions (≥1,150 mm^3^) showed a borderline association with diagnostic success (Table 5). Nodule density (solid vs. subsolid) and concentric CT-based localization were not associated with diagnostic yield, whereas a trend toward higher diagnostic performance was observed in the presence of a CT bronchus sign and when the lesion was visible on radial EBUS (Table 5). Anatomical location significantly influenced outcomes, with upper lobe lesions demonstrating higher diagnostic yield than lower lobe lesions. Procedural factors such as the use of cryobiopsy and PEEP level did not significantly affect diagnostic yield (Table 5).

In multivariate logistic regression analysis (Table 6), visibility of the lesion on augmented fluoroscopy (OR 3.22; 95% CI, 1.04–9.94; *p* = 0.043) and upper lobe location (OR 3.28; 95% CI, 1.12–9.65; *p* = 0.031) emerged as independent predictors of higher diagnostic yield, while other variables showed consistent positive trends without reaching statistical significance, underscoring the importance of intra-procedural imaging features in diagnostic performance.

A mixed-effects logistic regression sensitivity analysis demonstrated a low intraclass correlation coefficient (ICC = 0.021), indicating minimal clustering of lesions within patients. Upper lobe location, CABT visibility, and positive r-EBUS confirmation remained the strongest predictors of diagnostic success, with effect sizes comparable to those observed in the primary analysis.

### 3.6 Procedural safety and complications

The overall complication rate was 3.3% of procedures. All complications consisted of pneumothorax, none of which were associated with hemodynamic instability or major respiratory compromise. Chest tube placement was required in a limited number of cases.

No cases of major bleeding, respiratory failure, cardiac complications or procedure-related mortality were observed. Minor bleeding events were self-limiting and did not require additional intervention.

## 4 Discussion

The results of the present study confirm the effectiveness of shape-sensing robotic bronchoscopy combined with the LungVision digital tomosynthesis system for the diagnosis of peripheral pulmonary lesions (PPLs). The overall diagnostic yield of 74.5% falls within the range reported for advanced bronchoscopic navigation techniques based on augmented fluoroscopy, with values between 70 and 87% in major prospective studies (16–18). Compared with the series by Pritchett et al. (17), which reported a diagnostic yield of 70.6% for lesions <20 mm and 87% overall using LungVision, it should be noted that their cohort included larger nodules (median diameter 18 mm) and routinely used cone-beam CT (CBCT) confirmation, a potential confounder. Similarly, Cicenia et al. (16) primarily assessed navigation success rather than definitive diagnostic yield, which was approximately 75% and based on ROSE rather than final histology, while Pertzov et al. (18) reported a diagnostic yield of 77.8% (based on an intermediate definition of diagnostic yield) in lesions with a larger median diameter (25 mm). In contrast, the median nodule diameter in our study was 14 mm and the strict diagnostic yield reached 79.2% for lesions >1 cm and 87.5% for those >2 cm. These findings should be interpreted cautiously given the differences in lesion size, imaging confirmation strategies, and definitions of diagnostic yield among studies; nevertheless, our results should be interpreted as broadly consistent with prior series, while recognizing substantial methodological heterogeneity despite the smaller median lesion size observed in our cohort.

A major strength of this study lies in its novelty, as it represents—to our knowledge—the first real-world clinical evaluation of shape-sensing robotic-assisted bronchoscopy (ssRAB) combined with an AI-driven digital tomosynthesis platform (LungVision™ system). While both shape-sensing robotics and augmented fluoroscopy have independently demonstrated utility in peripheral lung navigation, evidence regarding their combined use remains limited. The rationale for this integration lies in the complementary strengths of these two technologies: robotic navigation ensures precise and stable access to peripheral airways, whereas digital tomosynthesis and augmented fluoroscopy provide real-time lesion localization and vital compensation for CT-to-body divergence. The favorable diagnostic yield observed in our cohort suggests that this technological synergy may significantly enhance targeting accuracy, particularly for small peripheral lesions.

Statistical analysis demonstrated that diagnostic yield is influenced by both pre-procedural and intra-procedural factors. In particular, upper lobe location and lesion visibility on C-arm based tomography (CABT) emerged as independent predictors of diagnostic success, highlighting the key role of real-time imaging in compensating for CT-to-body divergence, in line with findings by Pertzov et al. (18). Upper lobe location represents a favorable anatomical factor, likely due to reduced respiratory motion and improved mechanical stability compared with lower lobe lesions.

Other variables, including bronchus sign, radial EBUS identification, lesion size, and volume did not reach statistical significance but showed consistent positive trends and r-EBUS identification remained clinically relevant as an indicator of correct tool positioning. Notably, diagnostic yield in lesions without a bronchus sign (59%) was higher than that reported for conventional guided bronchoscopy (~49%) (19), confirming that robotic bronchoscopy may expand diagnostic indications even for lesions requiring transparenchymal approaches.

Importantly, this study includes the initial implementation phase of robotic bronchoscopy at our center, and a learning curve effect cannot be excluded. To explore the potential impact of the learning curve, we descriptively compared the first and second halves of the series (47 sampled lesions each). Median procedural time decreased from 42 min (IQR 30.5–53.5) to 38 min (IQR 30.5–47), while diagnostic yield increased from 72.3 to 76.6%. Although descriptive in nature, these findings suggest progressive optimization of procedural performance over time. This temporal trend should therefore be considered a potential confounding factor in the interpretation of the overall diagnostic yield. Existing evidence supports a relatively rapid learning curve and high reproducibility of robotic techniques (20). Overall, our findings are consistent with recent meta-analyses reporting pooled diagnostic yields between 69.6 and 87% (20–22), with comparable complication rates around 3% (21, 22). Beyond diagnostic performance, robotic bronchoscopy offers practical advantages, including short procedure time (34.5 min), low invasiveness and suitability for day-hospital settings, while maintaining a superior safety profile compared with transthoracic needle aspiration (8). Nevertheless, the high cost of robotic technology underscores the need for careful patient selection and future studies aimed at identifying refined eligibility criteria and predictors of success to optimize its clinical use.

This study demonstrates that robotic-assisted bronchoscopy combined with digital tomosynthesis is an effective modality for diagnosing peripheral pulmonary lesions, particularly in patients who are not candidates for more invasive procedures. The combined approach provided tissue suitable for molecular and immunohistochemical analysis in a substantial proportion of cases, supporting precision oncology through molecular profiling. However, NGS adequacy was not universal, as 5 of 14 requested cases were inadequate because of insufficient tumor cellularity and/or inadequate nucleic acid quality, highlighting an important limitation of bronchoscopic sampling for downstream molecular testing.

Furthermore, our findings should be contextualized within the rapidly evolving landscape of intra-procedural imaging technologies designed for peripheral bronchoscopy. Digital tomosynthesis (DT) and augmented fluoroscopy (AF) have emerged as highly accessible, cost-effective alternatives to fixed cone-beam CT (CBCT) for real-time target localization and mitigation of CT-to-body divergence. These tomographic reconstructions provide real-time 3D confirmation of tool alignment within the lesion, without the financial and radiation burdens associated with fixed CBCT (23). Recently, the field has moved toward fully integrated platforms. Notably, the prospective MATCH 2 study demonstrated that combining robotic bronchoscopy with embedded DT achieved a 93.5% tool-in-lesion rate on CBCT and a 96.7% strict diagnostic yield (24). Although single-platform integrated systems offer workflow advantages, our real-world data demonstrate that pairing an independent shape-sensing robot (Ion™) with an external DT/AF platform (LungVision™) supports the potential utility of real-time tomographic guidance even in a non-integrated platform configuration. In our multivariate analysis visibility on CABT emerged as a strong, independent predictor of diagnostic success (OR 3.22; *p* = 0.043). These findings support the potential relevance of real-time tomographic guidance in peripheral lung sampling, including within a non-integrated platform configuration, while acknowledging that direct comparative studies are needed to determine the relative contribution of imaging integration versus platform architecture.

Several limitations of this study warrant consideration. Firstly, the retrospective single-center design may limit the generalizability of the findings. Secondly, the absence of a direct comparator group, such as conventional bronchoscopy or CT-guided biopsy, prevents definitive assessment of the relative advantages of the approach studied. Thirdly, variability in operator experience could have influenced the outcomes, potentially limiting reproducibility in less experienced centers. Furthermore, although sampling tool selection was guided by institutional practice and recurrent procedural considerations, no fully standardized written decision algorithm was prospectively applied; this may represent a source of procedural heterogeneity and may limit reproducibility.

In addition, although a subset of patients contributed more than one lesion to the analysis, this potential source of non-independence is unlikely to have materially affected the main findings, as sensitivity analysis showed only a minimal within-patient clustering effect (ICC = 0.021).

While the sample size aligns with other real-world investigations, larger multicenter prospective trials are necessary to validate these results and refine patient selection criteria.

It is important to clarify that the results presented in this study pertain to strict diagnostic yield rather than diagnostic accuracy (that includes follow up data). Follow-up data for non-diagnostic cases are currently unavailable; therefore, false-negative rates, sensitivity, specificity, and overall diagnostic accuracy could not be determined. A comprehensive reassessment will be required once complete follow-up data are obtained to establish the true diagnostic performance of the robotic approach.

Cost-effectiveness analyses were beyond the scope of this study but remain an important consideration factor given the substantial economic investment required for robotic platforms.

### 4.1 Future perspectives

Future research should focus on comparative effectiveness studies evaluating robotic bronchoscopy against alternative diagnostic modalities across different patient populations and lesion characteristics.

Moreover, expanding the role of robotic platforms beyond diagnosis toward therapeutic interventions (25, 26)—such as localized ablation or bridge to surgery or radiation therapy via fiducial marker placement or local staining for fluorescence-guided approaches—represents a promising frontier in interventional pulmonology.

## 5 Conclusion

Robotic-assisted bronchoscopy combined with digital tomosynthesis appears to be a safe, effective, and reliable approach for the diagnosis of peripheral pulmonary lesions. The integration of advanced navigation technologies may significantly enhance lesion localization and diagnostic yield while maintaining a low complication rate. However, the absence of follow-up for non-diagnostic cases precludes assessment of false-negative rates and true diagnostic accuracy.

Our findings support the incorporation of this technology into the diagnostic workflow of specialized interventional pulmonology centers, particularly for patients requiring precise tissue sampling for oncologic decision-making. Further prospective studies are warranted to define optimal patient selection, cost-effectiveness and long-term clinical impact.

## Data Availability

The datasets presented in this study can be found in online repositories. The names of the repository/repositories and accession number(s) can be found in the article/supplementary material.
